# Geniposide-mediated protection against amyloid deposition and behavioral impairment correlates with downregulation of mTOR signaling and enhanced autophagy in a mouse model of Alzheimer's disease

**DOI:** 10.18632/aging.101759

**Published:** 2019-01-26

**Authors:** Zhihua Zhang, Xiaojian Wang, Di Zhang, Yueze Liu, Lin Li

**Affiliations:** ^1^ Key Laboratory of Cellular Physiology, Shanxi Medical University, Taiyuan, Shanxi, PR China; ^2^ Shanxi Medical College for Continuing Education, Taiyuan, Shanxi, PR China; ^3^ Shanxi Provincial People’s Hospital, Taiyuan, Shanxi, PR China; ^4^ Chemistry Department, Shanxi Medical University, Taiyuan, Shanxi, PR China; ^5^ Second Hospital, Shanxi Medical University, Taiyuan, Shanxi, PR China

**Keywords:** geniposide, APP/PS1 mice, mechanistic target of rapamycin, autophagy, Alzheimer’s disease

## Abstract

Geniposide, an iridoid glycoside extract from the gardenia fruit, is used in traditional Chinese medicine to alleviate symptoms of liver and inflammatory diseases. Geniposide activates GLP-1 receptors, known to modulate the activity of mechanistic target of rapamycin (mTOR), a key kinase regulating energy balance, proliferation, and survival in cells. mTOR activation inhibits autophagy, which is often disrupted in age-related diseases. Modulation of mTOR function to increase autophagy and inhibit apoptosis is involved in the protective effects of pharmacologic agents targeting diabetes and Alzheimer’s disease (AD). We investigated whether such mechanism could mediate geniposide’s neuroprotective effects in the APP/PS1 mouse model of AD. Eight-week treatment with geniposide improved cognitive scores in behavioral tests, reduced amyloid-β 1-40 plaque deposition, and reduced soluble Aβ1-40 and Aβ1-42 levels in the APP/PS1 mouse brain.This also showed increased p-Akt/Akt, p-mTOR/mTOR and decreased p-4E-BP1/4E-BP1 expression, and these patterns were partially reversed by geniposide. Evidence for enhanced autophagy, denoted by increased expression of LC3-II and Beclin1, was also seen after treatment with geniposide. Our data suggests that down regulation of mTOR signaling, leading to enhanced autophagy and lysosomal clearance of Aβ fibrils, underlies the beneficial effects of geniposide against neuropathological damage and cognitive deficits characteristic of AD.

## INTRODUCTION

Deposition of beta amyloid (Aβ) plaques in the brain is a main neuropathological hallmark of Alzheimer’s disease (AD), the most common neurodegenerative disorder worldwide. AD is progressive and so far irreversible, and is clinically characterized by cognitive decline. There is yet no pharmacological agent that can attenuate amyloid plaque formation or inhibit AD development once plaques started to form. type 2 diabetes mellitus (T2DM) is a risk factor for AD, and abnormal glucose metabolism is common to both conditions. The mechanistic target of rapamycin (mTOR) is a key kinase linking diabetes and AD. As a central integrator of nutrition sensing pathways, mTOR connects glucose and lipid metabolism to aging, and neuronal survival and degeneration by regulating energy balance in cells [1]. Overactivation of mTOR has been linked to insulin resistance, inflammation, and apoptosis. Its classical inhibitor, rapamycin, was shown to restore energy balance, promote autophagy and prevent apoptosis, induce immunosuppression, protect against neurodegeneration, and extend lifespan [2–4]. Given the close relationship between the pathophysiology of T2DM and AD, restoring glucose metabolism is a promising treatment strategy for AD [5]. Indeed, research has shown that some pharmacological agents used to treat T2DM could be beneficial for AD. Among them are the endogenous glucagon-like peptide-1 (GLP-1) peptide and its analogs [6–9], which modulate signaling through the PI3K/AKT/mTOR pathway. However, in pancreatic cells GLP-1 analogs activate mTOR, which is opposite to the main conclusion of this work, that the GLP-1 agonist geniposide inhibits mTOR. Because the pathways involved appear to be different in each case.

Gardenia jasminoides (Cape jasmine) is widely used in Chinese traditional medicine for the treatment of contusions, inflammation, brain disorders and hepatic disease [10, 11]. Geniposide, a monomeric glycoside extracted from the gardenia fruit, mediates several of its medicinal effects against inflammation [12], oxidative stress [13], diabetes [14] and asthma [15]. Because geniposide readily crosses the blood-brain barrier [16], studies have increasingly focused on its potential neuroprotective effects, especially on neurodegenerative disorders [17–19]. Using high-throughput screening, Liu et al. [20] identified geniposide as a GLP-1 receptor agonist. Gong et al. [21] demonstrated that geniposide acts through a GLP-1 receptor-dependent mechanism in vitro, which was shown in turn to mediate neurotrophic and neuroprotective effects in cultured neurons [22]. Also, Yin et al. reported that geniposide-activated phosphatidylinositol 3-kinase (PI3K)/Akt signaling mediated antioxidant protection in hippocampal neurons [23], and prevented Aβ_1-42_-induced toxicity in primary cultured cortical neurons [24]. Huang et al. [25] on the other hand, showed that geniposide protected neurons against post-ischemic neurovascular injury by regulating mTOR signaling.

Our past research showed, in turn, that geniposide attenuates memory impairment and reduces τ phosphorylation in the streptozotocin (STZ)-induced rat model of AD [18]. Given its protective effects in animal models of T2DM and AD [17], and based on its pharmacological actions on neurons, we hypothesize that geniposide downregulates mTOR activity to attenuate symptoms of AD. Using APP/PS1 mice to model human AD pathophysiology, in the present study we test the hypothesis that amyloid plaque reduction and cognitive improvement mediated by geniposide are correlated to mTOR inhibition and enhanced autophagy.

## RESULTS

### Geniposide ameliorates learning and memory impairments in APP/PS1 mice

**Figure 1:**
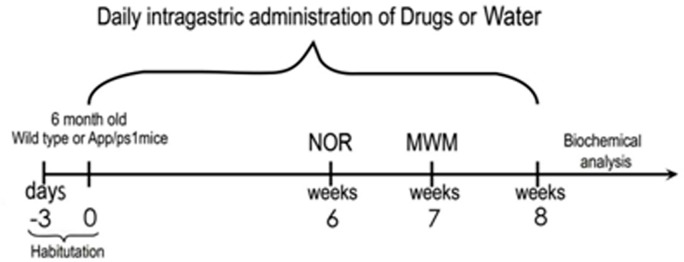
**Overview of the experimental design.** APP/PS1 and WT mice were treated with geniposide (50 mg/kg/d) or water, respectively, via intragastric administration every day for 8 weeks. The NOR test was conducted in the sixth week, and the MWM test was conducted in the seventh week. On week eight mice were killed for biochemical analyses.

**Figure 2:**
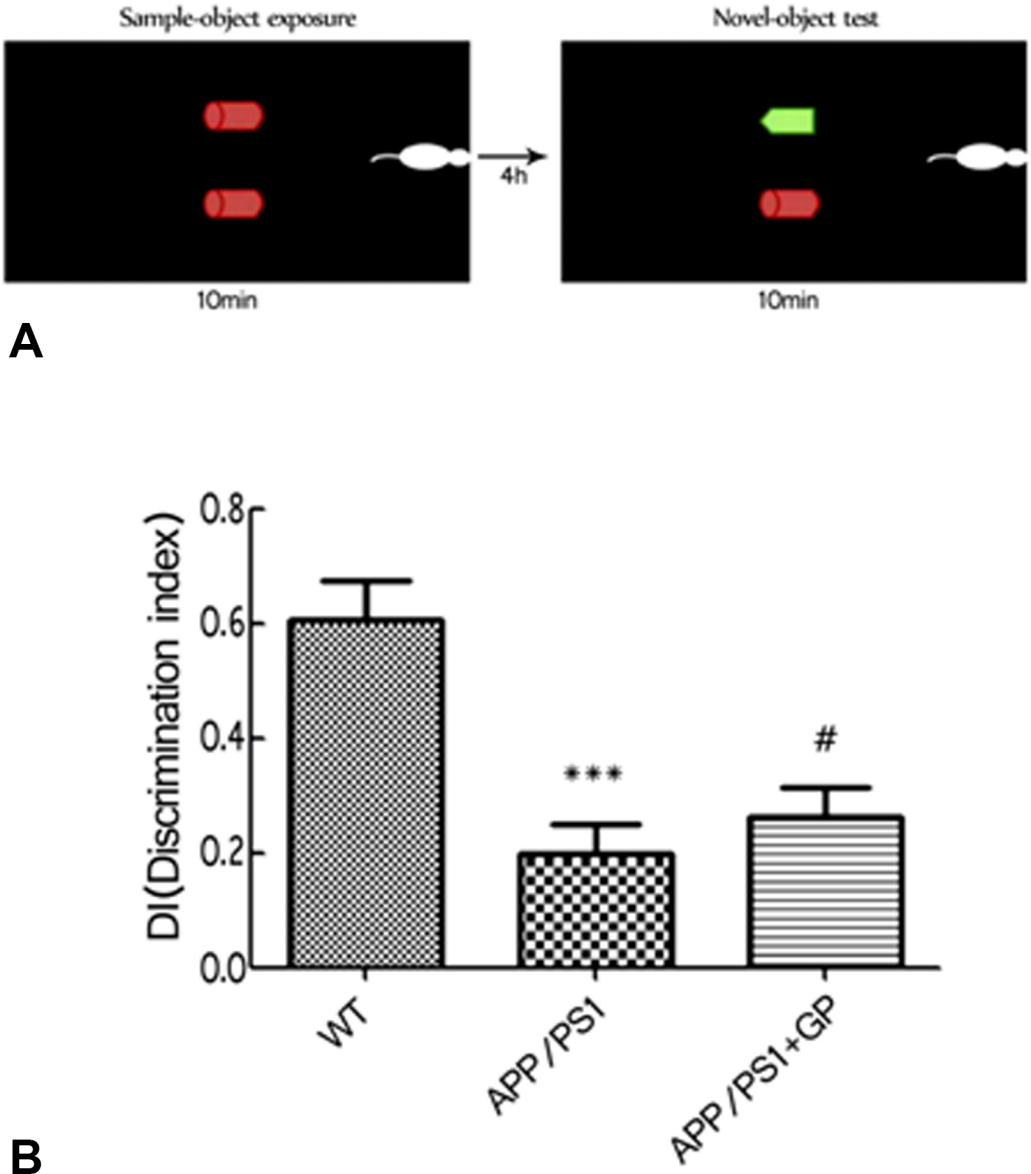
**Geniposide improves NOR scores in APP/PS1 mice.** (**A**) Schematic diagram of the NOR test. (**B**) NOR test results. The DI of APP/PS1 mice was significantly decreased compared to WT, and was improved by geniposide treatment. Data are mean ± SEM (n = 13–15). ^***^p < 0.001 vs. WT; ^#^*p* < 0.05 vs. APP/PS1. (one-way ANOVA, Tukey's Multiple Comparison Test). WT: wild-type mice. GP: geniposide.

Novel object recognition (NOR) and Morris water maze (MWM) tests were conducted to assess whether geniposide administration could attenuate behavioral deficits in APP/PS1 mice. APP/PS1 double-transgenic mice (n = 15) were given a daily intragastric geniposide dose (50 mg/kg/d) for a total of 8 weeks (including the last two involving behavioral tests). Controls included cohorts of APP/PS1 mice (n = 15) and age-matched C57BL/6 wild-type (WT) mice that were given water instead (Figure 1) [26]. Results of the NOR test showed significant cognitive impairment in untreated APP/PS1 mice, reflected by a discrimination index (DI) of 0.198 ± 0.004 vs 0.607 ± 0.005 in WT mice (*p* < 0.001). After geniposide treatment, significant improvement was detected in APP/PS1 mice (0.263 ± 0.004; *p* < 0.05 compared to untreated APP/PS1 mice) (Figure 2B).

**Figure 3:**
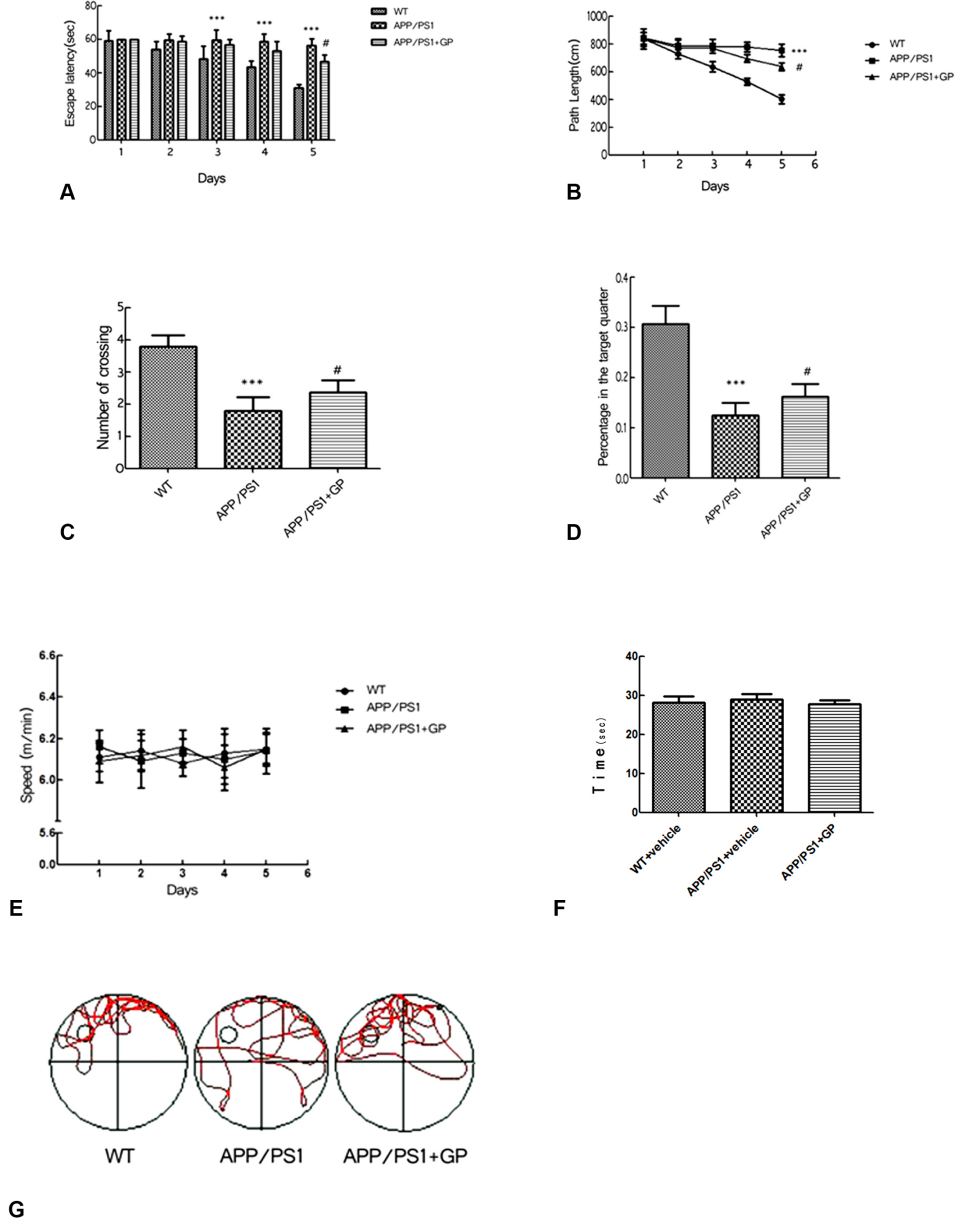
**Geniposide improves learning and memory in APP/PS1 mice.** (**A**) Escape latency in the MWM’s place navigation test was significantly longer in APP/PS1 mice compared to WT on days 3–5, and shortened by day 5 in mice treated with geniposide. (**B**) Path length (swimming distance) was longer in APP/PS1 mice than in WT mice on days 3–5, and shortened by day 5 in geniposide-treated mice. (**C**) The number of crossings over the area where the escape platform was previously located (spatial probe test) was decreased in APP/PS1 mice compared to WT. This decrease was partly reversed after geniposide treatment. (**D**) The time spent in the target quadrant was decreased in APP/PS1 mice compared to WT, and this was partly improved by geniposide. (**E**) Swimming speed did not differ between groups. (**F**) Swimming time to arrive at visible platform did not differ between groups. (**G**) Swimming tracks. Data are presented as mean ± SEM (n = 13–15). ***p < 0.001 vs. WT; ^#^*p* < 0.05 vs. geniposide-treated APP/PS1 mice (two-way ANOVA, Tukey's Multiple Comparison Test). WT: wild-type mice. GP: geniposide.

Learning and memory functions were further evaluated using two versions of the MWM test. Over the course of the place navigation test (i.e. 5 consecutive days), escape latency became progressively shorter in WT mice but remained unchanged in untreated APP/PS1 mice. In APP/PS1 mice treated with geniposide, however, signifiacant reductions in escape latency (46.58 ± 12.27 s vs 56.17 ± 6.73 s in untreated APP/PS1 mice; *p* < 0.05) (Figure 3A) and swimming path length (635 ± 23.62 cm vs 750 ± 26.76 cm in untreated APP/PS1 mice, *p* < 0.05) were recorded on test day 5 (Figure 3A–3B).

Next, memory retrieval ability was evaluated after the escape platform was removed from the water tank (spatial probe test). By tracking swimming patterns, we recorded the number of crossings over the previous platform location, the percentage of time spent in the area (maximum 60s), and swimming speeds. The two first parameters were significantly lower in APP/PS1 mice than in WT mice (1.780 ± 0.770 vs 3.800 ± 0.330 crossings, *p* < 0.001, and 13% ±0.061% vs 31% ±0.036%, *p* < 0.001).

Again, improvements were observed in geniposide-treated mice, i.e. higher number of crossings, and more time spent in the target quadrant (2.19 ± 0.21 and 0.16 ± 0.03%, respectively; both *p* < 0.05 compared to untreated APP/PS1 mice) (Figure 3C–3D). There were no significant differences in swimming speed (Figure 3E) and time to arrive at the visible platform (Figure 3F) between the three groups.

Swimming tracks are shown in Figure 3G. In APP/PS1 mice those were disorganized, indicating that the mice sought the hidden platform randomly. In contrast, geniposide-treated APP/PS1 mice stayed longer in the target area and showed a more selective search track.

### Geniposide attenuates brain histopathology in APP/PS1 mice

**Figure 4:**
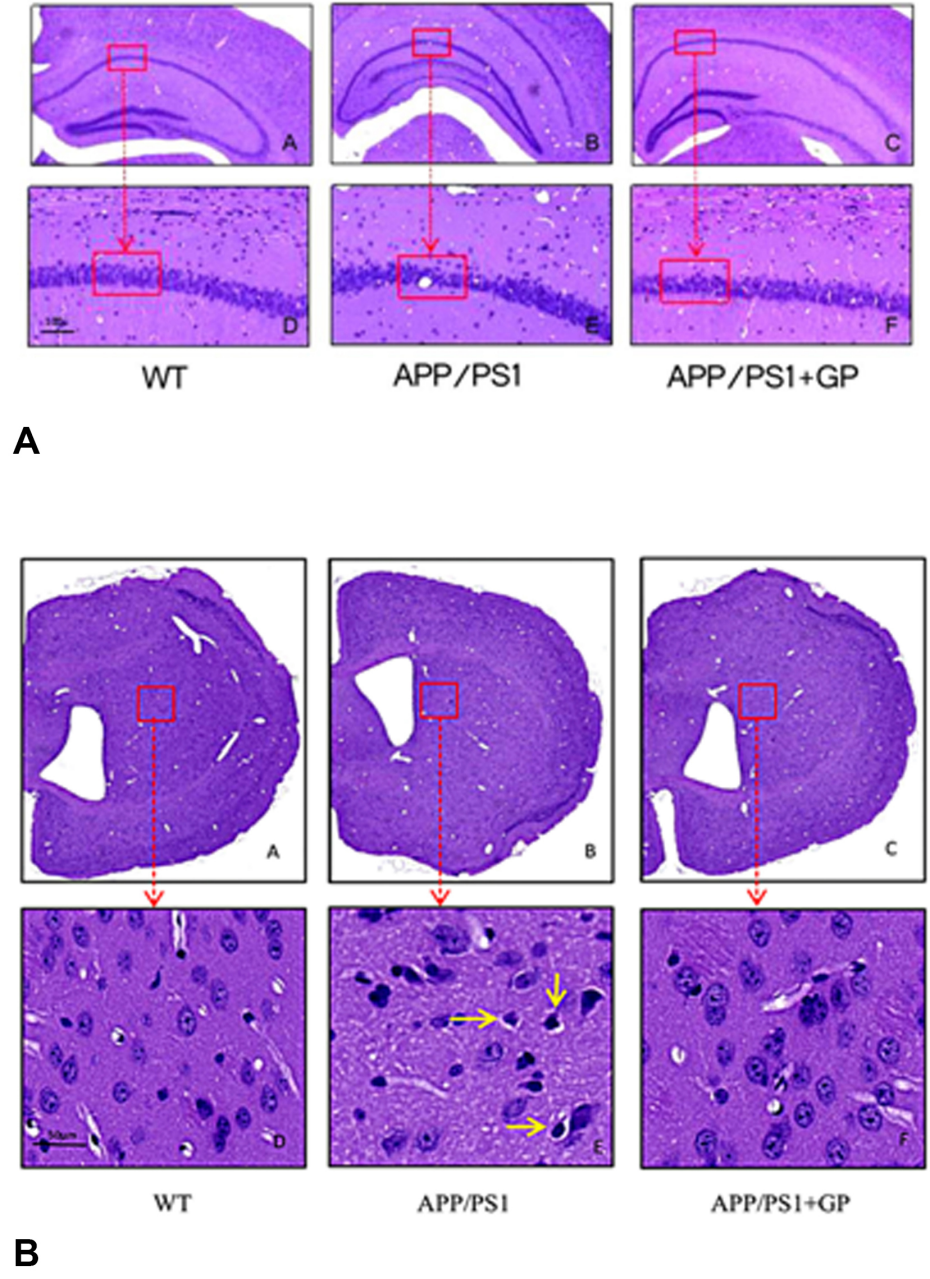
**Geniposide ameliorates neuropathological changes in APP/PS1 mice.** Sections obtained from the hippocampus (**A**) and cortex (**B**) were stained with HE. Neurons in WT mice were normal in shape and orderly arranged. In APP/PS1 mice, neurons were lost and darkly stained. Shrinkage and necrosis were observed in scattered neurons (yellow arrow). Geniposide treatment reduced these pathological changes. A) A-C, 4× magnification; D-F, 20× magnification; B) A-C, 2× magnification; D-F, 40× magnification).

Whole brain coronal sections were cut to analyze whether cytopathological changes in the cortex and hippocampus of APP/PS1 mice could be attenuated by geniposide. The number of neurons was reduced and their contents concentrated with deep staining and surviving neurons was shrinkage and necrosis in APP/PS1 mice. All of these changes were significantly ameliorated by geniposide (Figure 4A–4B).

### Geniposide reduces brain Aβ 1-40 plaque formation in APP/PS1 mice

**Figure 5:**
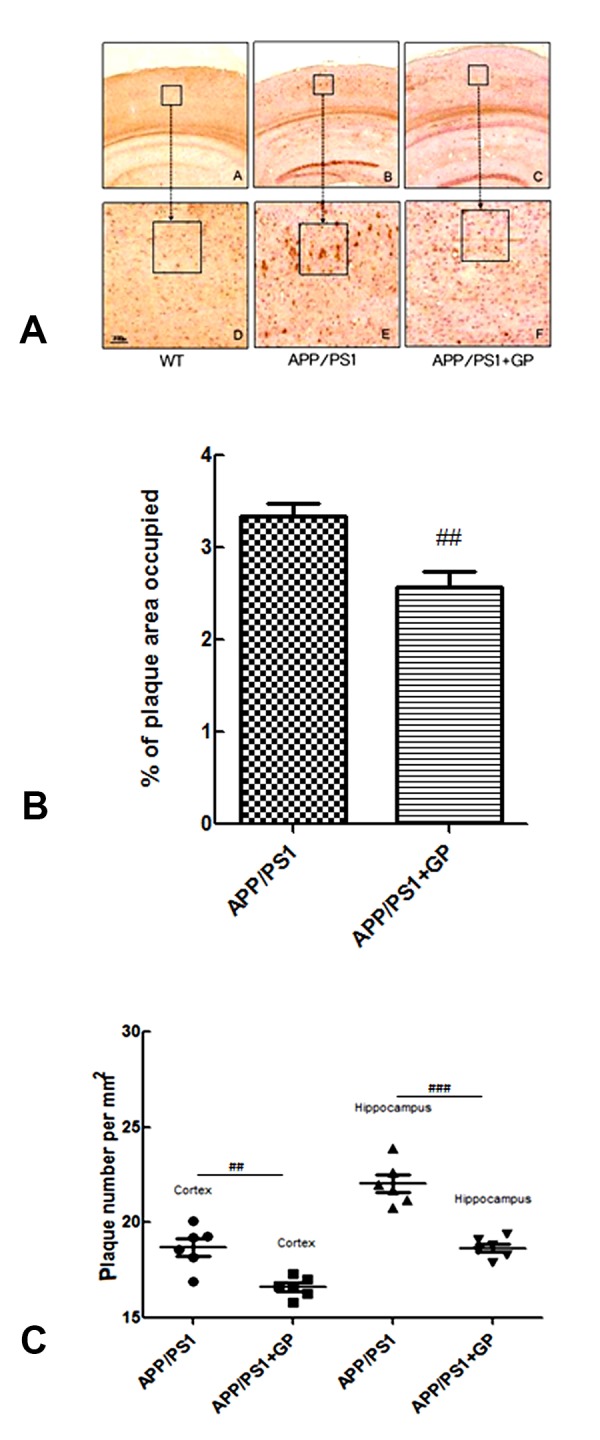
**Geniposide attenuates Aβ1-40 plaque formation in the hippocampi of APP/PS1 mice.** (**A**) Representative images of Aβ1-40-stained brain sections (A-C: 4× magnification; D-F: 10× magnification). (**B**) Percentage of the area occupied by Aβ 1-40 plaques. (**C**) Quantification of Aβ plaque density. Data are presented as mean ± SEM (n = 6). ^##^*p* < 0.01, ^###^*p* < 0.001 (one-way ANOVA, Tukey's Multiple Comparison Test). WT: wild-type mice. GP: geniposide.

Senile plaques including Aβ fibrils are one of the neurological hallmarks of AD. We used immunohistochemistry to investigate the potential of geniposide to reduce brain Aβ plaque formation in APP/PS1 mice. As shown in Figure 5A, APP/PS1 mice had characteristically abundant Aβ1-40plaque deposition throughout the brain. Treatment with geniposide reduced the area occupied by Aβ1-40 plaques (2.545 ± 0.342% vs 3.317 ± 0.457%, ,*p* < 0.01; Figure 5B), as well as their density (16 ± 2.4 vs 20.4 ± 2.8 plaques/mm^2^, *p* < 0.05; Figure 5C), compared to untreated APP/PS1 mice.

### Geniposide decreases soluble Aβ1-40 and Aβ1-42 levels in the hippocampi of APP/PS1 mice

**Figure 6:**
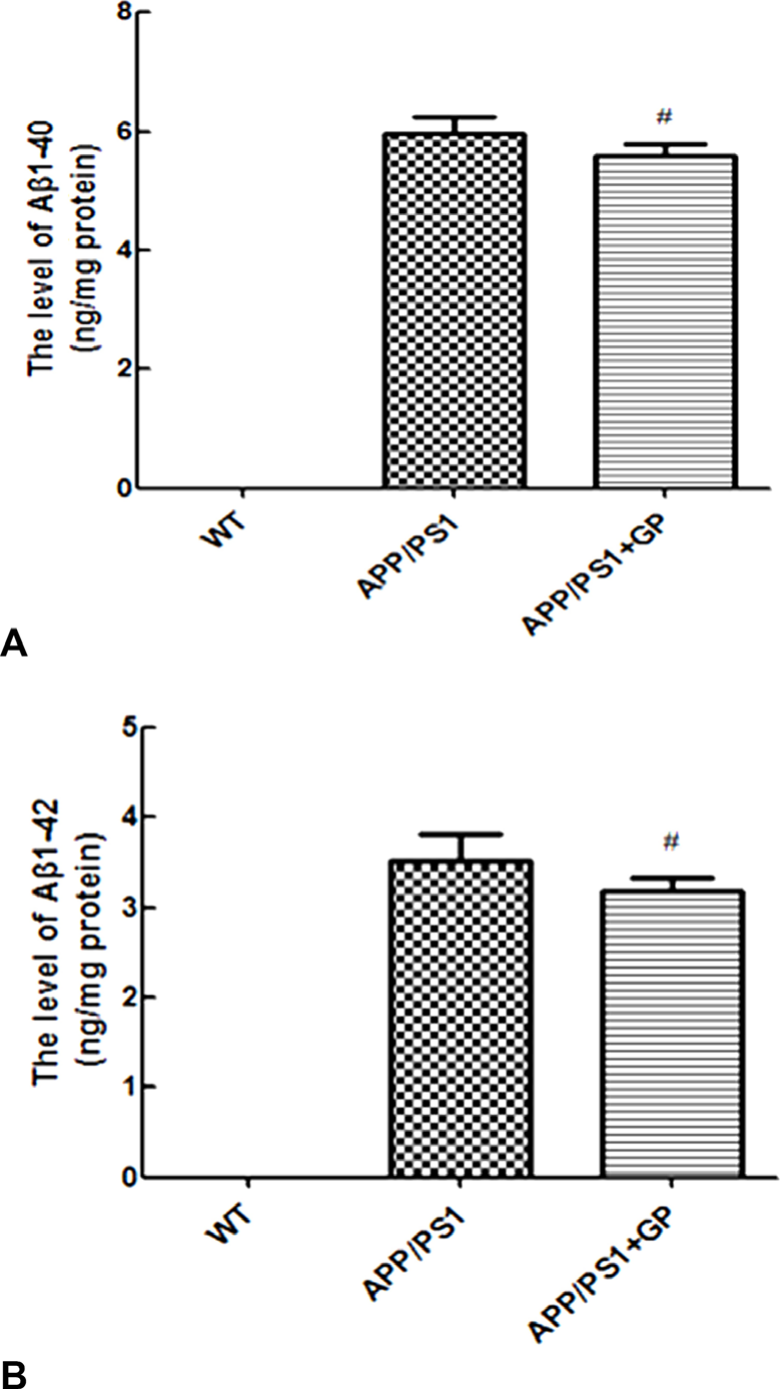
**Geniposide reduces soluble Aβ1-40 and Aβ1-42 levels in hippocampi of APP/PS1 mice.** Soluble Aβ1-40 (**A**) and Aβ1-42 (**B**) levels were measured by ELISA in the hippocampi of WT and APP/PS1 mice. In the latter, geniposide treatment lowered the expression of both Aβ alloforms. Data are presented as mean ± SEM (n = 6). ^#^*p* < 0.05 (one-way ANOVA, Tukey's Multiple Comparison Test). WT: wild-type mice. GP: geniposide.

To test whether decreased plaque formation correlated with reduced Aβ synthesis, we further measured hippocampal levels of soluble Aβ oligomers (Aβ1-40 and Aβ1-42) using ELISA. Consistent with our Aβ1-40 immunostaining findings, soluble Aβ1-40 and Aβ1-42 levels were elevated in untreated APP/PS1 mice (5.97 ± 0.04 and 3.52 ± 0.04 ng/mg protein, respectively), and decreased (5.58 ± 0.03 and 3.18 ± 0.02 ng/mg protein; *p* < 0.05) after treatment with geniposide (Figure 6A–6B).

### Geniposide up-regulates brain expression of LC3-II and Beclin1 in APP/PS1 mice

**Figure 7:**
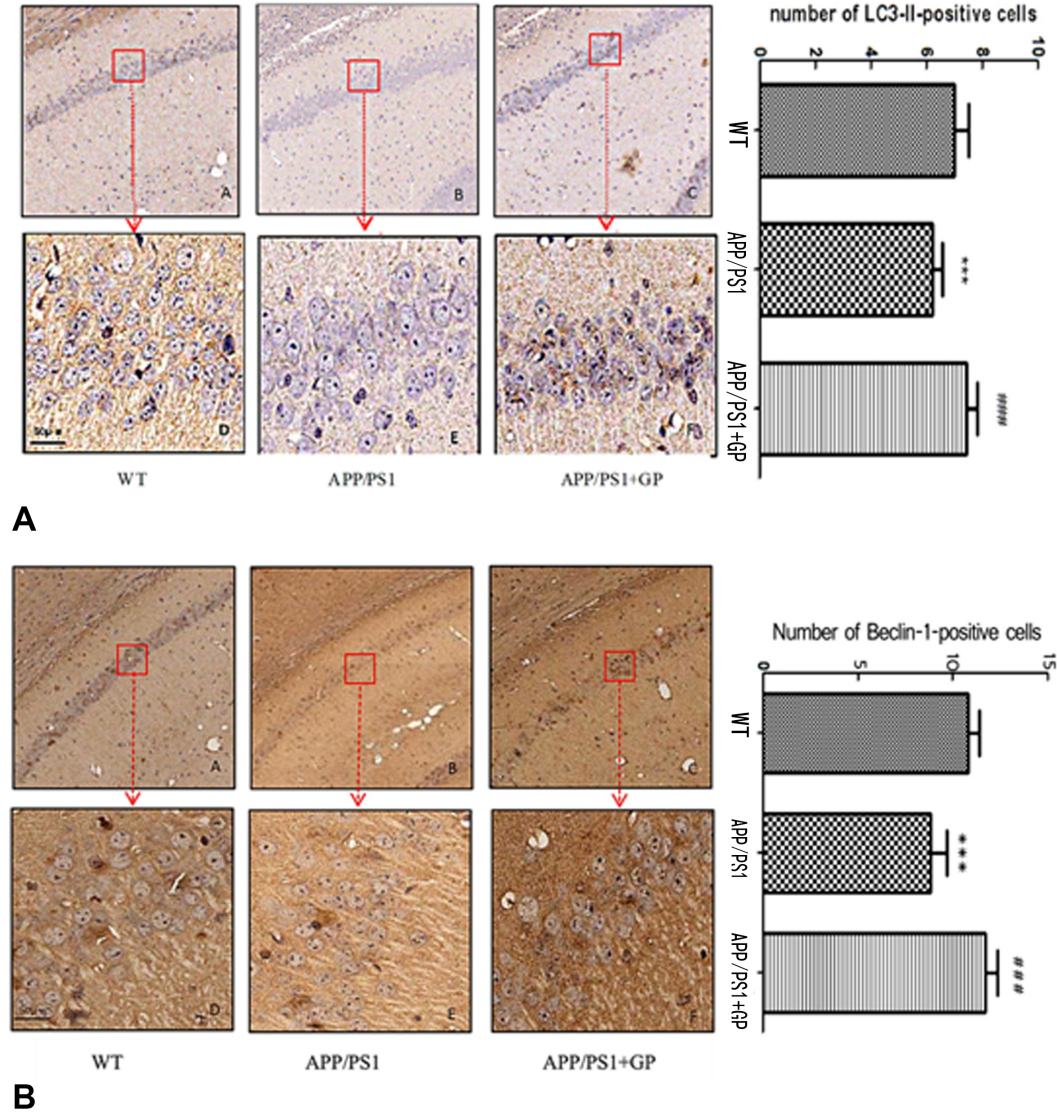
**Geniposide upregulates the expression of autophagy markers in the hippocampi of APP/PS1 mice.** (**A**) Representative images of LC3-II-stained brain sections and quantification of LC3-II-positive neurons; (**B**) Representative images of Beclin1-stained brain sections and quantification of Beclin1-positive neurons. Numbers of both LC3-II and Beclin1 positive neurons in APP/PS1 mice were reduced compared to WT, and geniposide reversed those reductions. (A-C, 4× magnification; D-F, 40× magnification). Data are presented as mean ± SEM (n = 6). *** p < 0.001, APP/PS1 mice vs. WT; *^###^ p* < 0.001, geniposide-treated vs. untreated APP/PS1 mice (one-way ANOVA, Tukey's Multiple Comparison Test). WT: wild-type mice. GP: geniposide.

To explore potential molecular mechanisms underlying geniposide’s neuroprotective effects in APP/PS1 mice, we first measured the expression of two autophagy-related markers, LC3-II and Beclin1, using immunohistochemistry. As shown in Figure 7A, fewer LC3-II-positive neurons were present in hippocampi of APP/PS1 mice compared to WT, and this reduction was reversed by geniposide. Similarly, geniposide treatment restored the expression of Beclin1, which was found to be decreased in untreated APP/PS1 mice (Figure 7B).

The number of LC3-II- and Beclin1-positive neurons was reduced, respectively, from 7.12 ± 2.05 and 10.22 ± 1.35 neurons/mm^2^ in WT mice to 5.40 ± 0.93 and 8.86 ± 1.17 neurons/mm^2^ in APP/PS1 mice (*p* < 0.001 for both comparisons), and increased after geniposide treatment to 8.61 ± 1.74 `and 10.65 ± 1.08 respectively (*p* < 0.001 for both comparisons, in relation to untreated APP/PS1 mice) (Figure 7A–7B).

### Geniposide reduces the expression of mTOR pathway activation markers in APP/PS1 mice

**Figure 8:**
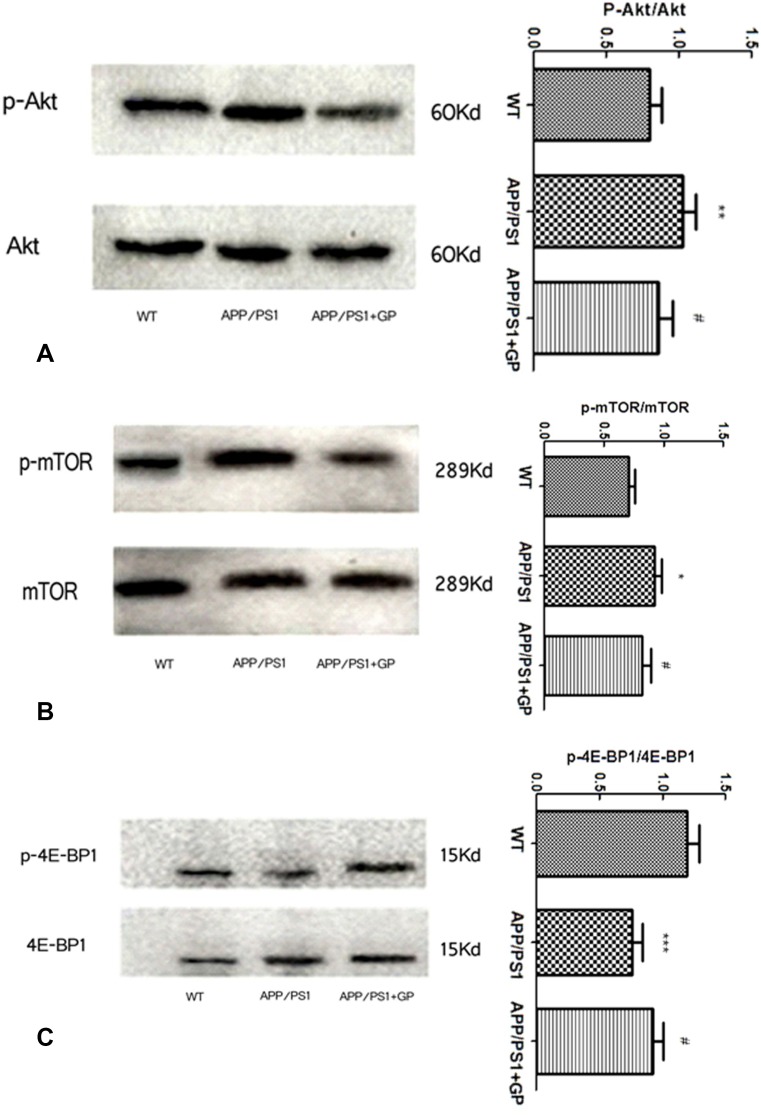
**Geniposide treatment decreases mTOR activation markers in brains of APP/PS1 mice.** Hippocampal expression of Akt, mTOR, and 4E-BP1, and their respective phosphorylated forms was detected by western blot. The expression of p-Akt (**A**) and p-mTOR (**B**) was enhanced in APP/PS1 mice compared to WT, and geniposide attenuated this increase. The expression of p-4E-BP1 (**C**) in APP/PS1 mice was reduced compared to WT, and geniposide partly restored this decrease. Data are presented as mean ± SEM (n = 6). ***p < 0.001, **p < 0.01, *p < 0.05 vs. WT; ^#^*p* < 0.05 vs. APP/PS1 mice (one-way ANOVA, Tukey's Multiple Comparison Test). WT: wild-type mice. GP: geniposide.

To evaluate potential modulation of mTOR pathway-related proteins by geniposide, expression of Akt, mTOR, and 4E-BP1, and their phosphorylated forms, was measured using western blot. As shown in Figure 8A, brain p-Akt levels were increased in APP/PS1 mice (*p* < 0.01 compared to WT), and geniposide blunted this increment (*p* < 0.05). Similarly, brain p-mTOR expression was higher in APP/PS1 than in WT mice (*p* < 0.05) and was attenuated by geniposide (*p* < 0.05) (Figure 8B). In contrast, p-4E-BP1 levels were decreased in APP/PS1 mice compared to WT (*p* < 0.001), and partially restored in mice treated with geniposide (*p* < 0.05) (Figure 8C).

## DISCUSSION

The present study provides evidence that geniposide, a natural compound derived from the Gardenia fruit that acts as a GLP-1 agonist, attenuates amyloid plaque formation and cognitive impairment in the APP/PS1 mouse model of AD. These effects were correlated with decreased levels of the phosphorylated forms of AKT and mTOR, and increased levels of the autophagy markers LC3-II and Beclin1. These data suggest that inhibition of AKT/mTOR might underlie the protective actions of geniposide in animal models of AD [27].

Aging is a key risk factor for AD [2, 28]. In fact, some of the neuropathological processes seen during AD also occur in the brain during normal aging. The mTOR pathway influences neuronal development and plasticity, and its dysfunction in the aging brain may contribute to cognitive decline [29–32]. Inhibition of mTOR was shown to reverse learning deficits in Tsc2+/− mice [33], and memory deficits induced by delta-9-tetrahydro-cannabinol in normal mice [34]. Given the pleiotropic effects of mTOR on cellular activity extensive research is being conducted on mTOR inhibitors as tools to treat not only neurodegenerative disorders, but also a wide range of metabolic disorders such as T2DM, cancer, and cardiovascular disease [35, 36].

Current evidence suggests a tight relationship between mTOR, Aβ production, and AD [35]. Activation of mTOR increased amyloid precursor protein synthesis and deposition, in part by inhibiting autophagy-mediated Aβ clearance [3], while restoring autophagy through inhibition with rapamycin reversed cognitive decline and ameliorated Aβ pathology [37].

Autophagy is essential for cell survival, proliferation, and homeostasis and is also a key process connecting mTOR and pathomolecular changes in AD. Decreased nutrient availability and calorie restriction inhibit mTOR and promote autophagy, improving cell survival and prolonging lifespan via the breakdown of cell constituents into amino acids and other small molecules, which reenter anabolic pathways [38–41]. Wang et al. 42] showed that curcumin, a polyphenol isolated from the turmeric root, inhibited the expression of both PI3K and phosphorylated Akt, attenuated cognitive impairments, and inhibited Aβ generation in APP/PS1 mice. Meanwhile, Tramutola er al. [43] analyzed post-mortem samples of brain tissue from AD patients and proposed that altered mTOR signaling and autophagy occur during the early stages of AD, and that hyperactivation of the PI3K/Akt/mTOR pathway is synchronous with autophagy impairment. Our study shows that the autophagy-related proteins LC3-II and Beclin1 are downregulated in the brains of APP/PS1 mice, and geniposide increased their levels in parallel with a decrease in p-Akt and p-mTOR, and an increase in p-4E-BP1, a substrate of mTOR.

These data support the hypothesis that geniposide-mediated inhibition of mTOR improves autophagic function and ameliorates AD-related molecular processes (Aβ accumulation) and behavioral deficits in APP/PS1 mice. Previous evidence hinted at the molecular bases of the interplay between autophagy, Aβ production and clearance, and their control by mTOR. Jiang et al. [44] showed that the mTOR inhibitor temsirolimus enhanced Aβ clearance in HEK293-APP695 cells and in the brains of APP/PS1 mice in an autophagy-dependent manner. They also observed cognitive improvements, and decreased cellular apoptosis in the hippocampi of temsirolimus-treated APP/PS1 mice. Yin et al. [24] showed that pre-incubation of primary cultured cortical neurons with geniposide prevented Aβ1-42-induced toxicity, and increased secretion of insulin-degrading enzyme (IDE), a major Aβ-degrading protease. Interestingly, Son et al. [45] showed in turn that rapamycin increased IDE secretion from astrocytes, in a process regulated by the autophagy pathway.

Two GLP-1 analogs currently used to treat T2DM, namely exendin-4 (exenatide) and liraglutide, were tested in clinical trials in AD patients. Exendin-4 treatment for 18 months (trail NCT01255163) increased nausea and failed to improve cognitive measures, although the number of participants was low. A second trial (NCT01469351) showed that liraglutide treatment for 6 months did not attenuate amyloid deposition nor improved cognitive scores, although it increased cerebral glucose metabolic rate (cGMR) in non-diabetic AD patients [46]. A third study, the Evaluating Liraglutide in Alzheimer's Disease (ELAD) trial (NCT01843075), is currently in the recruitment phase. These relatively short-term studies present however some caveats. It would be of much interest, for instance, to evaluate whether long-term treatments of diabetic patients with GLP-1 agonists reduce the risk of AD. The present study and previous, related evidence suggest that geniposide might be a natural, safe, and effective agent to prevent AD symptoms when administered early to at-risk individuals.

## MATERIALS AND METHODS

### Chemicals

Geniposide (purity> 98%) was obtained from MedChem Express. All antibodies, including anti-Aβ1-40, anti-LC3-II, anti-Beclin1, anti-Akt, anti-p-Akt, anti-mTOR, anti-p-mTOR, anti-4E-BP1, and anti-p-4E-BP1, were purchased from Bioworld Technology Inc. (Nanjing, China). ELISA kits for Aβ1-40 and Aβ1-42, and BCA protein assay reagents were purchased from Boster Biotechnology Co., Ltd. (Wuhan, China). All chemicals were of reagent grade.

### Animals and drug administration

Experimental APP/PS1 double-transgenic mice and C57BL/6 background mice (6 months old, males) were obtained from Beijing HFK Bio-Technology Co., Ltd. APP/PS1 mice resemble well the physiopathology of human disease and develop obvious amyloid plaque deposition in the brain at 6 months of age. The animals were individually maintained in plastic cages at 23 ± 1°C with humidity of 55% ± 5% based on a 12h light-dark cycle. All mice were allowed free access to food and water. Before experiments, all mice were housed for 3 days to adapt to the environment.

Mice were randomly assigned to one of 3 groups: (i) APP/PS1 double-transgenic mice (n = 15) fed with geniposide (50 mg/kg/d); (ii) APP/PS1 double-transgenic mice (n = 15) fed with an equal volume of water [26]; and (iii) Age-matched C57BL/6 wild-type (WT) mice (n = 12) fed with water as control. Geniposide and water were administrated intragastrically once a day for 8 weeks, including the 2 weeks of behavioral testing. The experimental design is summarized in Figure 1.

All animal procedures were performed in accordance with the guidelines for Care and Use of Laboratory Animals (NIH Publications No.8023, revised 1996), and the study protocol was reviewed and approved by the Shanxi Medical University Laboratory Animals Care and Use Committee.

### Novel object recognition test

The novel object recognition (NOR) test was used to assess learning and memory in mice, as it mimics the learning and memory processes of human behavior [47]. The test is based on the spontaneous interest displayed by mice in exploring a new object, rather than a familiar one. The test had 3 phases: in the habituation phase, each mouse was allowed to roam freely on an open-field area (an empty black box 60 cm wide × 60 cm deep × 60 cm high) for 10 min; in the familiarization phase, 2 identical red wood cylinders (A1 and A2) were presented for 10 min; after 24 h, one of these cylinders was replaced by a novel object, a same-sized plastic green cylinder (B), after which recognition memory (the time spent exploring each object) was recorded.The discrimination index (DI) was calculated as (N-F) /(N+F) × 100%, where N = time spent exploring the novel object (green cylinder), and F = time spent exploring the familiar object (red cylinder).

### Morris water maze (MWM) tests

The equipment consisted of a stainless-steel circular water tank (120 cm in diameter and 50 cm in height) with its inner wall painted white. An escape platform (14 cm in diameter) made invisible by tinting the water with titanium dioxide was located 1.5 cm underwater. Water temperature was kept at 25 ± 2°C during the test. Swimming trajectories were recorded by a video camera system (EthoVision 3.0, Noldus Information Technology, Wageningen, Holland).

### *Place navigation test*

The ability of mice to learn and access memory was assessed using the place navigation test, conducted on 5 consecutive days. The water tank was divided into four quadrants and four fixed points were selected as starting points. A mouse was placed in the water at one of the four points and allowed to swim freely to find the hidden platform. Escape latency, i.e., the time to reach the platform, and path length (swimming distance) were recorded. If the mouse failed to find the platform within 60s, the experimenter guided it onto the platform for 5s and the escape latency recorded was 60s.

### *Spatial probe test*

The spatial probe test of memory retrieval ability was performed on the 6^th^ experimental day, i.e., a day after conclusion of the place navigation test. The hidden platform was removed, and a mouse was placed in the water tank with its face towards the wall in a randomly selected quadrant. The number of crossings over the area the platform’s previous location, and the percentage of time spent in the target quadrant (up to 60s) were recorded.

### *Visible platform test*

To exclude the potential effect of motor ability and visual impairments on the experimental results, a visible platform test was performed following the spatial probe test. The time required for the mice to find the visible platform and their average swimming speed were recorded.

### Immunohistochemical staining

Following behavioral tests, the mice were transcardially perfused with 0.9% saline and 4% paraformaldehyde under anesthesia with chloral hydrate (350 mg/kg). The brains were quickly removed, fixed in 4% paraformaldehyde, dehydrated in ethanol and embedded in paraffin for HE and immunohistochemical staining.

Brain sections were deparaffinized with xylene and graded ethanol solutions and treated with 3% H_2_O_2_ for 10 min to inactivate endogenous peroxidase. Anti-Aβ1-40 antibody, and antibodies against two autophagy markers, microtubule-associated protein 1 light chain 3 (LC3)-II and Beclin1, were applied (1:200) at 4°C overnight. After washing, the sections were incubated with biotinylated goat anti-mouse secondary antibody at room temperature for 20 min, followed by 20 min treatment with an avidin-biotin peroxidase complex reagent. Peroxidase activity was visualized with 3,3-diaminobenzidine (DAB), and sections were counterstained with hematoxylin. For quantitative analysis, six sections of the same brain region were selected from each mouse, and photomicrographs were captured under an optical microscope. Aβ1-40 plaques were quantitatively analyzed by estimating: a) percentage of stained area; and b) plaque density, expressed as the number of Aβ1-40 plaques per mm^2^.

### Quantitation of soluble Aβ1-40 and Aβ1-42 levels by ELISA

The expression of Aβ1-40 and Aβ1-42 in the hippocampus was further quantified using an enzyme-linked immunosorbent assay (ELISA), purchased from Bioworld Technology Inc. (Nanjing, China), in accordance to the manufacturer’s protocols. Hippocampi were homogenized in 5M guanidine hydrochloride using a hand-held homogenizer, and the homogenates were centrifuged at 20,000 × g at 4°C for 30 min. Aβ1-40 and Aβ1-42 levels were quantitatively assessed from the samples’ supernatants.

### Western blot

The hippocampus and cortex were dissected and preserved at -80°C for western-blot analyses. Tissues were lysed in cold RIPA buffer containing complete protease and phosphatase inhibitors. Homogenates were centrifuged at 4°C and 15,000×g for 10 min, and protein concentrations determined with a BCA protein assay. Western blot analyses followed standard procedures. Protein samples (40-60 μg) were run on 8%, 10%, or 12% SDS-PAGE gels and transferred onto PVDF membranes. The membranes were blocked in 5% bovine serum albumin in TBST (Tris-buffered saline with 0.05% Tween-20) for 1h, and incubated overnight at 4°C with primary antibodies directed against: Akt (1:1,000), p-Akt (1:2,000), mTOR (1:1,000), p-mTOR (1:1,000), 4E-BP1 (1:1,000), or p-4E-BP1 (1:2,000), followed by incubation at 4°C for 2h with a goat-anti-rabbit IgG-horseradish peroxidase-conjugated secondary antibody (Boster, Wuhan, China).

Immunoreactive bands were visualized using ECL-enhanced chemiluminescence (Boster Biotechnology Co., Ltd. Wuhan, China), captured with a chemiluminescence imaging system (Sage Creation, Beijing, China), and digitalized using Quantity One 4.31 software (Bio-Rad, Hercules, CA, USA).

### Statistical analysis

Data are presented as mean ± SEM. Statistical analysis was carried out using GraphPad Prism 5 (Graph-Pad software Inc., San Diego, CA, USA). *p*< 0.05 was considered significant. Date obtained from MWM experiments were analyzed by two-way ANOVA with repeated measures. Comparisons among different groups were made by one-way ANOVA and Tukey’s Post Test.

## ETHICS STATEMENT

This study was approved by the Shanxi Medical University ethics committee and all investigators complied with ethical standards.
